# Noninvasive quantification of alveolar morphometry in elderly never- and ex-smokers

**DOI:** 10.14814/phy2.12583

**Published:** 2015-10-13

**Authors:** Gregory A Paulin, Alexei Ouriadov, Eric Lessard, Khadija Sheikh, David G McCormack, Grace Parraga

**Affiliations:** 1 Imaging Research Laboratories, Robarts Research Institute, The University of Western OntarioLondon, Ontario, Canada; 2 Department of Medical Biophysics, The University of Western OntarioLondon, Ontario, Canada; 3 Division of Respirology, Department of Medicine, The University of Western OntarioLondon, Ontario, Canada

**Keywords:** Acinar duct, emphysema, hyperpolarized ^3^He magnetic resonance imaging, lung morphometry, senile emphysema

## Abstract

Diffusion-weighted magnetic resonance imaging (MRI) provides a way to generate in vivo lung images with contrast sensitive to the molecular displacement of inhaled gas at subcellular length scales. Here, we aimed to evaluate hyperpolarized ^3^He MRI estimates of the alveolar dimensions in 38 healthy elderly never-smokers (73 ± 6 years, 15 males) and 21 elderly ex-smokers (70 ± 10 years, 14 males) with (*n *=* *8, 77 ± 6 years) and without emphysema (*n *=* *13, 65 ± 10 years). The ex-smoker and never-smoker subgroups were significantly different for FEV_1_/FVC (*P *=* *0.0001) and DL_CO_ (*P *=* *0.009); while ex-smokers with emphysema reported significantly diminished FEV_1_/FVC (*P *=* *0.02) and a trend toward lower DL_CO_ (*P *=* *0.05) than ex-smokers without emphysema. MRI apparent diffusion coefficients (ADC) and CT measurements of emphysema (relative area–CT density histogram, RA_950_) were significantly different (*P *=* *0.001 and *P *=* *0.007) for never-smoker and ex-smoker subgroups. In never-smokers, the MRI estimate of mean linear intercept (260 ± 27 *μ*m) was significantly elevated as compared to the results previously reported in younger never-smokers (210 ± 30 *μ*m), and trended smaller than in the age-matched ex-smokers (320 ± 72 *μ*m, *P *=* *0.06) evaluated here. Never-smokers also reported significantly smaller internal (220 ± 24 *μ*m, *P *=* *0.01) acinar radius but greater alveolar sheath thickness (120 ± 4 *μ*m, *P *<* *0.0001) than ex-smokers. Never-smokers were also significantly different than ex-smokers without emphysema for alveolar sheath thickness but not ADC, while ex-smokers with emphysema reported significantly different ADC but not alveolar sheath thickness compared to ex-smokers without CT evidence of emphysema. Differences in alveolar measurements in never- and ex-smokers demonstrate the sensitivity of MRI measurements to the different effects of smoking and aging on acinar morphometry.

## Introduction

Senile emphysema – the normal changes of the lung parenchyma that accompany aging – is characterized by distal airway enlargement without obvious fibrosis or alveolar wall destruction (Verbeken et al. 1992). Other structural components include the loss of elastic fibers, thickening of alveolar walls (Verbeken et al. 1992), and diminished pulmonary elastic recoil (Frank et al. 1957; Thurlbeck 1967; Turner et al. 1968). In concert with the pathological changes that accompany aging, increased residual volume (RV), functional residual capacity (FRC) (Janssens et al. 1999), and decreased diffusing capacity of carbon monoxide (DL_CO_) (Janssens et al. 1999), forced expiratory volume in 1 sec (FEV_1_), and forced vital capacity (FVC) (Fletcher and Peto 1977) are also observed.

Senile emphysema in elderly never-smokers is not commonly accompanied by clinical symptoms or pulmonary function measurements typical of smoking-related emphysema (Laennec and Forbes 1834). Emphysema that commonly accompanies chronic obstructive pulmonary disease (COPD) may be differentiated from the lung changes associated with aging by the deformation of alveoli as a result of fibrosis and tissue destruction, resulting in reduced surface area for gas exchange (Hogg 2004). Importantly, older adults typically report lung function that deteriorates with age, but in the elderly, such normal (age-normalized) lung function is sufficient for routine day-to-day activities (Mayer et al. 1958). However, there is an increased risk of breathlessness and respiratory failure in the elderly, and these may further complicate other comorbidities of aging (Peterson et al. 1981; Young et al. 1987; Sharma and Goodwin 2006). In addition, age-dependent lung structural and functional differences can reduce the sensitivity of the respiratory centers in the presence of hypoxia or hypercapnia, resulting in a diminished ventilatory response in cases of heart failure or aggravated airway obstruction (Kronenberg and Drage 1973; Peterson et al. 1981; Janssens et al. 1999).

Hyperpolarized inhaled noble gas magnetic resonance imaging (MRI) provides noninvasive, in vivo measurements of lung function and structure (Yablonskiy et al. 2002; Fain et al. 2005; Evans et al. 2007; Parraga et al. 2007; Kirby et al. 2010) showing those regions of the lung that participate in ventilation and those that do not (Parraga et al. 2008; Kirby et al. 2010). In addition, the MRI apparent diffusion coefficient (ADC) for inhaled gases is sensitive to changes in the lung microstructure and airspace size correlating well with age (Fain et al. 2005), spirometry measurements (Salerno et al. 2002), DL_CO_ (Fain et al. 2006), and X-ray computed tomography (CT) measurements of emphysema (Diaz et al. 2009). Previous studies have also shown the strong agreement for alveolar parameters obtained using ^3^He MRI and those estimated using histology (Yablonskiy et al. 2009). The relationships between MRI estimates of the mean linear intercept and pulmonary function measurements have also been shown in mild to severe cases of COPD (Woods et al. 2006; Yablonskiy et al. 2009; Quirk et al. 2011).

On the basis of the previous work, we hypothesized that elderly ex- and current smokers would report significantly increased external airway radius (*R*) and mean linear intercept (*L*_m_), compared to elderly never-smokers. Therefore, the aim of this work was to use MRI to provide ADC and acinar/alveolar morphometry estimates in elderly never-smokers and ex-smokers as a first step toward understanding lung aging in relation to smoking history and other measurements of pulmonary function.

## Methods

### Study volunteers and design

Participants provided written informed consent to a study protocol approved by the local research ethics board and Health Canada. Never-smokers aged 60–90 years with ≤0.5 pack–years smoking history and without acute or chronic respiratory disease, as well as smokers aged 60–90 years with >10 pack-years smoking history and were evaluated using spirometry, plethysmography, hyperpolarized ^3^He MRI, and CT during a single 2-h visit.

### Pulmonary function measurements

Spirometry was performed to acquire the forced expiratory volume in 1 sec (FEV_1_), forced vital capacity (FVC), and FEV_1_/FVC according to American Thoracic Society (ATS) guidelines (MedGraphics Corporation, St. Paul, Minnesota) (Miller et al. 2005). Body plethysmography was performed for the measurement of lung volumes, and DL_CO_ was measured using the gas analyzer (MedGraphics, St. Paul, MN).

### Image acquisition

MRI was performed on a whole body 3 T MRI system (MR750 Discovery, GEHC, Milwaukee, WI) with broadband imaging capability. All ^3^He MRI employed a whole body gradient set with maximum gradient amplitude of 4.8 G/cm and a single-channel, rigid elliptical transmit/receive chest coil (RAPID Biomedical GmbH, Wuerzburg, Germany). The basis frequency of the coil was 97.3 MHz and excitation power was 2 kW using an AMT 3T90 RF power amplifier (GEHC). Subjects were positioned supine in the scanner and for both ^1^H and ^3^He MRI, subjects were instructed by a pulmonary function technologist to inhale of 1.0 L ^3^He/N_2_ a gas mixture (20%/80% by volume) from functional residual capacity (FRC), with image acquisition performed under breath-hold conditions as described previously (Parraga et al. 2007). Diffusion-weighted ^3^He MRI data were acquired using a multislice interleaved 2D gradient echo diffusion-weighted sequence with a matrix size of 128 × 80, for seven 30-mm coronal slices (900 *μ*sec selective RF pulse, flip angle *θ *= 4°, TE = 3.9 msec, TR = 5.6 msec, bandwidth = 62.5 kHz, *b *=* *0, 1.6, 3.2, 4.8, 6.4 sec/cm^2^); the diffusion-sensitization gradient pulse ramp up/down time was 500 *μ*sec with a diffusion time of 1460 *μ*sec. The potential for image artifacts associated with RF pulse “history” (Miller et al. 2004) was addressed by using an optimal constant flip angle of 4 degrees (Ouriadov et al. 2009). A diffusion-sensitizing, gradient-step, k-space acquisition scheme starting at the maximum *b* value was used to ensure that maximum MR signal was acquired for diffusion-weighted images at greater *b* values. All five *b*-value images were acquired during a single 15 sec breath-hold.

Thoracic CT was acquired on a 64-slice Lightspeed VCT scanner (GEHC) (64 × 0.625 mm, 120 kVp, 100 effective mA, tube rotation time of 500 msec, and a pitch of 1.0). A single spiral acquisition of the entire lung was acquired from the apex to the base with subjects in the supine position and in breath-hold after inhalation of a 1.0 L ^4^He/N_2_ mixture from FRC. Images were reconstructed using a slice thickness of 1.25 mm with a standard convolution kernel. The total effective dose for an average adult was 1.8 mSv.

### Image analysis

Ventilation defect percent (VDP) measurements were generated by one observer using semiautomated segmentation software as described previously (Kirby et al. 2012). ^3^He MRI ADC analysis was performed using MATLAB R2013b (MathWorks, Natick, MA). To ensure that ADC values were generated for voxels corresponding to ventilated lung regions, a k-means clustering algorithm (Kirby et al. 2012) was applied to the nondiffusion-weighted images (*b *=* *0 s/cm^2^) to obtain a binary mask for each slice. The resulting binary masks were then applied to the corresponding diffusion-weighted images (*b *=* *1.6 sec/cm^2^), and ADC maps were generated on a voxel-by-voxel basis as described previously (Yablonskiy et al. 2002).

The minimum signal-to-noise ratio (SNR) of 40 (Ouriadov et al. 2014) for the *b *=* *0 sec/cm^2^ image and the minimum SNR of 5 for the *b *=* *6.4 sec/cm^2^ image were used as thresholds for the generation of morphometric estimates. For each subject, a single region-of-interest (ROI) (approximately 100 voxels) inside the lung was used to obtain the mean signal value for SNR measurements. A single ROI outside of the lung was used to estimate the signal in regions of the image with mainly noise. The standard deviation of the signal value measured outside the lung was used to estimate noise (approximately 100 voxels) and SNR was calculated based on the following equation:

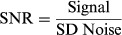
1

A Hann filter was applied to maximize SNR of images. The SNR threshold of 40 for *b* = 0 images was used as described previously (Ouriadov et al. 2013) to mitigate potential errors in the anisotropic diffusion coefficient estimations and consequently, errors in the morphometric parameters. The SNR threshold of 5 for *b* = 6.4 sec/cm^2^ images was used because this is the minimum threshold acceptable for quantitative image analysis (Rose 1948).

The relative area of the CT density histogram with attenuation values less than −950 Hounsfield units (RA_950_) was determined using Pulmonary Workstation 2.0 (VIDA Diagnostics Inc., Coralville, IA). The CT density threshold for RA_950_ greater than 6.8% was used (Gevenois et al. 1996) to classify smokers with and without emphysema.

### Lung morphometry calculations and estimates

**Figure 1:**
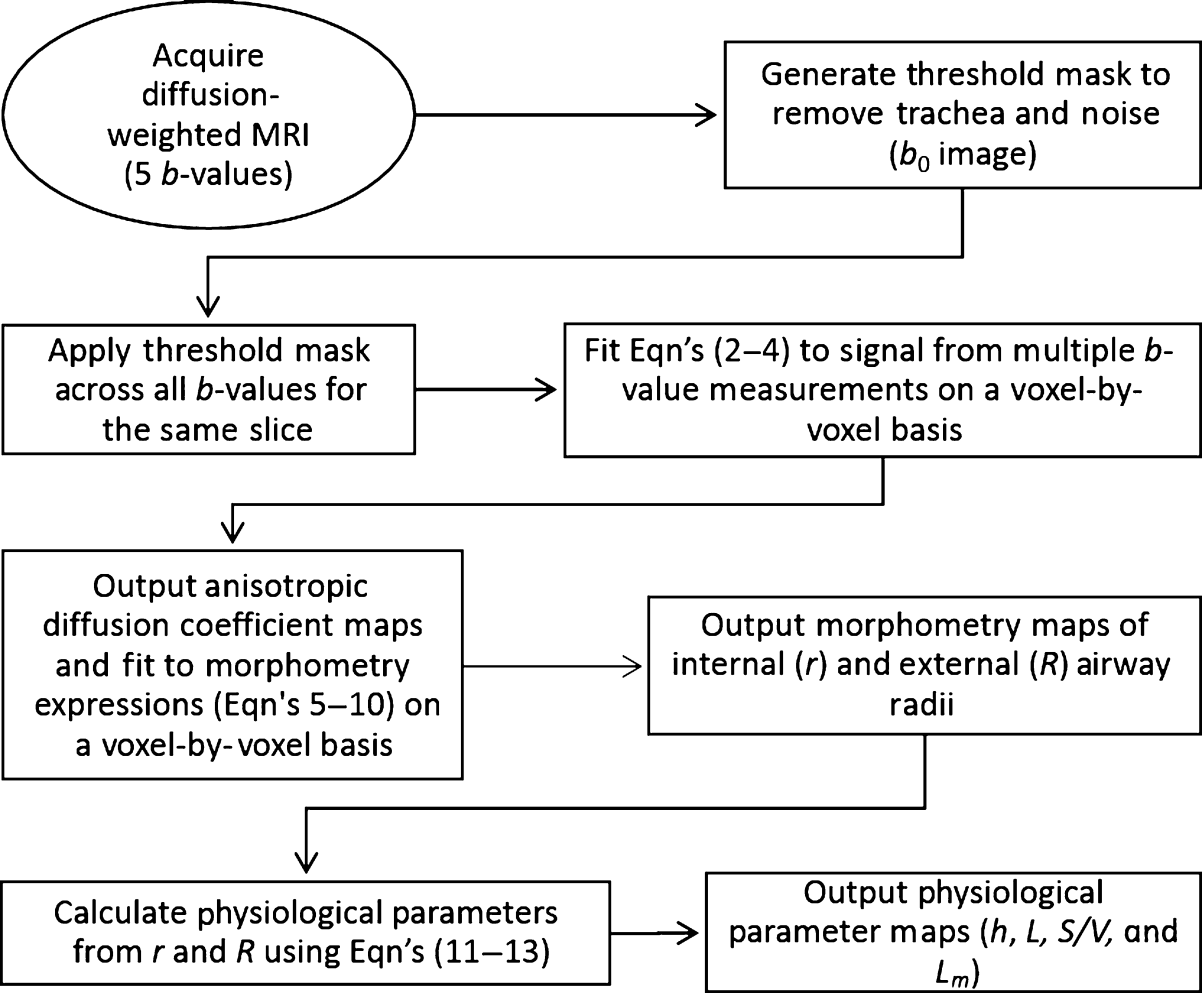
Schematic for pipeline required to derive morphological parameters from ^3^He diffusion-weighted MRI. Anisotropic diffusion coefficient maps were generated using a custom-built IDL 6.4 algorithm which fit equations 2–4 to multiple *b*-value measurements of the ^3^He diffusion-attenuated MR signal on a voxel-by-voxel basis. Anisotropic diffusion coefficients were substituted using phenomenological expressions 5–10 in order to generate morphometry maps: internal (*r*) and external (*R*) airway radius. Alveolar depth (*h*), alveolar length (*L*), surface area-to-volume ratio (*S/V*), and mean linear intercept (*L*_*m*_) were calculated on a voxel-by-voxel basis using morphometry map data in equations 11–13.

A schematic for the MRI morphometry data generation is provided in Figure1. As described previously (Sukstanskii and Yablonskiy 2008), anisotropic diffusion coefficient maps were generated using a custom-built IDL 6.4 algorithm which fit equation 2 to multiple *b*-value measurements of the ^3^He diffusion-attenuated MR signal on a voxel-by-voxel basis (Ouriadov et al. 2013, 2015) with the assumption of constant *D*_L_ and *D*_T_ values. The same ADC binary masks were applied to the corresponding diffusion-weighted images prior to fitting. Using this approach, *S*_0_ is the MR signal intensity in the absence of diffusion-sensitizing gradients, *Φ(z)* is the error function 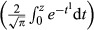), *D*_*L*_ is the longitudinal diffusion coefficient, and *D*_*T*_ is the transverse diffusion coefficient.

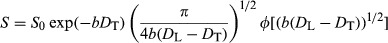
2

Equations 2–4 were used to calculate the geometrical expressions for internal (*r*) and external (*R*) airway radius, using equations 5–10 developed previously (Yablonskiy et al. 2002, 2009) on a voxel-by-voxel basis using previously published fitting algorithm (Ouriadov et al. 2014, 2015), where *R*, *r*, and *D*_0_ were the fitting variables. For a physiological range of geometrical parameters *r* and *R* (*r/R *>* *0.4) (Yablonskiy et al. 2009), and for gradient strengths typical of clinical scanners, the following equations may be used (Sukstanskii and Yablonskiy 2008):

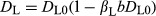
3

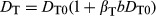
4

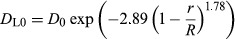
5

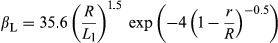
6

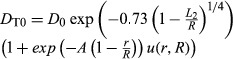
7

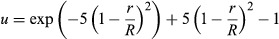
8

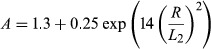
9

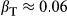
10

which account for non-Gaussian diffusion in acinar airways, where *β*_L_ and *β*_T_ are the coefficients that reflect non-Gaussian diffusion effects (Sukstanskii and Yablonskiy 2008), *D*_L0_ and *D*_T0_ are anisotropic diffusion coefficients at *b *=* *0 sec/cm^2^, where *L*_1_ and *L*_2_ are the characteristic diffusion lengths for one- and two-dimensional diffusion (*L*_1_ = 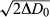 and *L*_2_ = 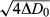) and *D*_0_ is the unrestricted diffusion coefficient for ^3^He in the gas mixture. Previous simulations showed that 1.6–1.8 msec is the optimal diffusion time to permit alveolar measurements in mild and moderate COPD (Yablonskiy et al. 2009). Two conditions also determine the maximum *b* value (Yablonskiy et al. 2009) such that *D*_L_ and *D*_T_ can be estimated if: (1) 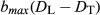 > 1; and (2) *b*_*max*_ *D*_*T*_ > 1, that is, a maximum *b* value should be greater than 6 sec/cm^2^. Finally, parameters such as alveolar depth (*h*), alveolar length (*L*), surface area-to-volume ratio (*S/V*), and mean linear intercept (*L*_m_) were calculated on a voxel-by-voxel basis by fitting morphometry map data into equations 11 through 13 (Yablonskiy et al. 2009).

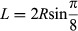
11

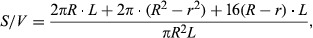
12

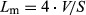
13

### Statistical analysis

Independent *t*-tests, tests for normality (determined with a Shapiro–Wilk test), and analysis of variance with post hoc analysis using the Holm–Bonferroni correction were performed using SPSS Statistics, V22.0 (SPSS Inc., Chicago, IL). For measurements that were not normally distributed, multiple comparisons were evaluated using the Kruskal–Wallis test with Dunn’s correction. The relationships between morphometry and spirometry measurements were evaluated using Pearson correlations performed using GraphPad Prism 4.01 (GraphPad Software, La Jolla, CA; 2004). Results were considered statistically significant when the probability of making a Type I error was less than 5% (*P* < 0.05).

## Results

### Demographics and pulmonary function measurements

As shown in Table1, 59 participants were enrolled including 38 never-smokers (73 ± 6 years, 15 males) and 21 ex-smokers (70 ± 10 years, 14 males) all of whom provided written informed consent to an ethics board approved protocol. Table S1 (online only) provides a by-subject list of all demographic data that are summarized in Table1. There were significant differences observed between never- and ex-smokers for FEV_1_ (*P* = 0.02), FEV_1_/FVC (*P* = 0.0001), RV/TLC (*P* = 0.01), and DL_CO_ (*P* = 0.002) but not for age, BMI, FVC, or TLC. Ex-smokers were classified based on the presence or absence of emphysema measured using the CT RA_950_ threshold described previously (Gevenois et al. 1996) and there was no significant difference in smoking history between the two subgroups (ex-smokers = 40 ± 21 pack-years, ex-smokers with emphysema = 44 ± 31 pack-years, ex-smokers without emphysema = 38 ± 12 pack-years; unadjusted *P* = 0.62). The average years since smoking ceased for all ex-smokers was 17 ± 12 years (ex-smokers with emphysema = 20 ± 14 years and ex-smokers without emphysema = 15 ± 12 years; unadjusted *P* = 0.47). Ex-smokers with emphysema reported a trend toward diminished FEV_1_/FVC (*P* = 0.05) and a trend toward abnormal DL_CO_ (*P* = 0.05) compared to ex-smokers without emphysema. As shown in Table S1, two never-smokers reported FEV_1_/FVC <0.70 and four ex-smokers without emphysema also reported FEV_1_/FVC <0.70 and FEV_1_ consistent with GOLD grade I (*n* = 2) or grade II (*n* = 2) COPD.

**Table 1:** Participant demographics

Parameter (mean ± SD)	All (*n* = 59)	Never-smokers (*n* = 38)	Ex-smokers (*n* = 21)			Significant differences (*P* value)			
			All (*n* = 21)	ES (*n* = 8)	ESnE (*n* = 13)	NS-S	NS-ES	NS-EnE	ES-EnE
Male sex, *n* (%)	33 (56)	15 (39)	14 (66)	7 (88)	7 (54)	0.28	0.08	1	0.81
Age, years	72 (8)	73 (6)	70 (10)	78 (6)	66 (10)	1	0.95	0.09	**0.02**
BMI, kg/m^2^	27 (4)	26 (3)	28 (5)	27 (3)	30 (6)	0.92	1	0.31	0.99
FEV_1%pred_	100 (24)	110 (17)	87 (29)	72 (34)	97 (22)	**0.02**	**0.02**	0.51	1
FVC_%pred_	100 (18)	100 (15)	99 (22)	94 (26)	100 (19)	1	0.89	1	1
FEV_1_/FVC %	72 (11)	77 (5)	64 (13)	52 (11)	71 (8)	**0.0001**	**<0.0001**	0.17	0.05
RV %_pred_	110 (31)*	100 (21)	120 (39)§	150 (50)	110 (17)¶	**0.02**	**0.001**	1	0.15
TLC %_pred_	100 (13)*	100 (13)	110 (14)§	110 (15)	100 (13)¶	1	0.56	1	1
RV/TLC %_pred_	100 (21)*	95 (15)	110 (26)§	130 (32)	100 (14)¶	**0.01**	**0.003**	0.69	0.42
DL_CO_ %_pred_	83 (20)†	90 (17)‡	71 (21)§	55 (16)	82 (17)¶	**0.009**	**0.0003**	1	0.05

Significant differences (*P* value) generated using a Kruskal–Wallis test with Dunn’s correction. Bold values denotes significant difference (*P* < 0.05).

SD, standard deviation; BMI, body mass index; FEV_1_, forced expiratory volume in 1 sec; %pred, percent predicted; FVC, forced vital capacity; RV, residual volume; TLC, total lung capacity; DL_CO_, diffusing capacity of the lung for carbon monoxide.

* *n* = 58

† *n* = 55

‡ *n* = 36

§ *n* = 20

¶ *n* = 12.

### Imaging measurements

**Figure 2:**
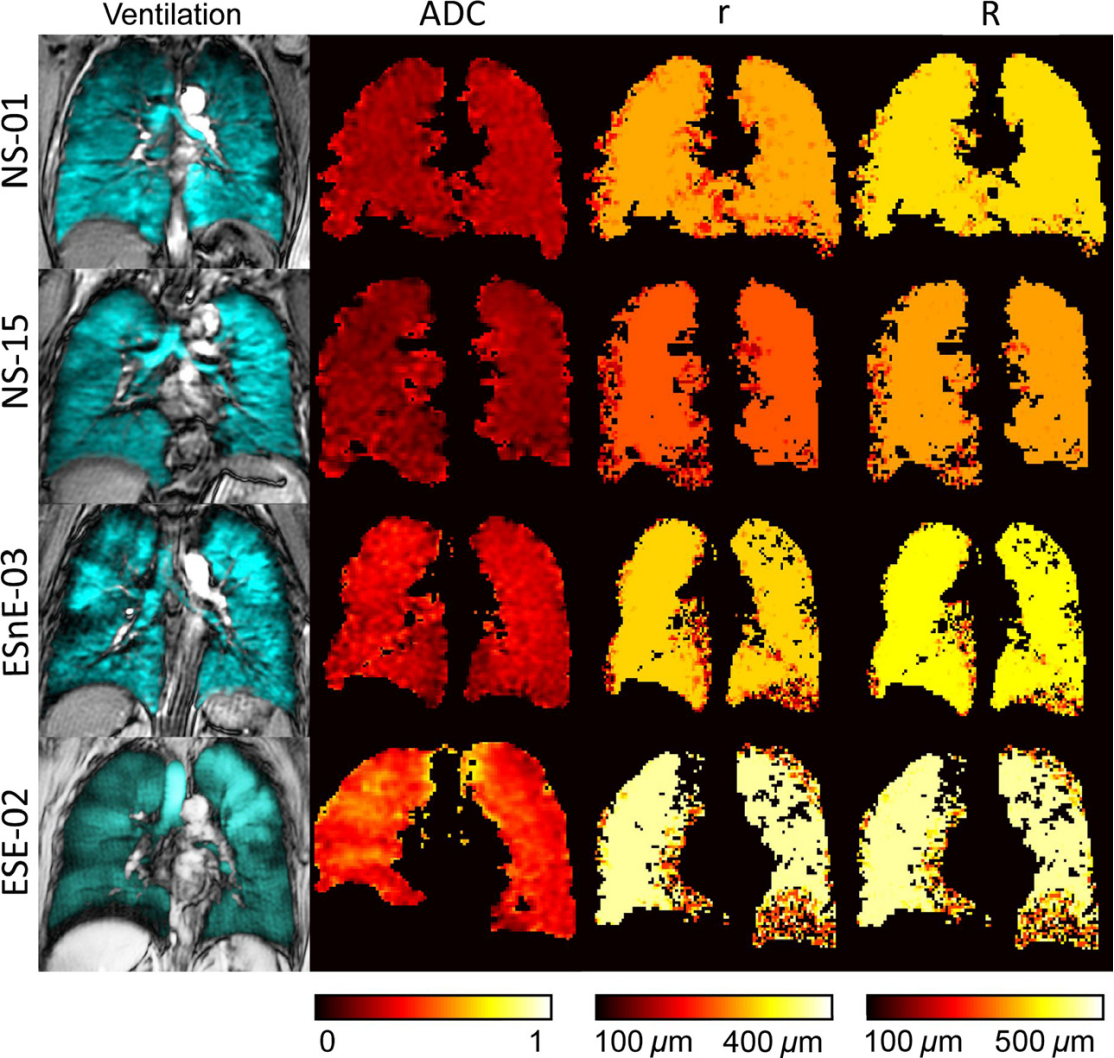
^3^He center slice static ventilation, ADC, and morphometry maps. Hyperpolarized ^3^He static ventilation, ADC, and morphometry maps for two representative older never-smokers and two ex-smokers. ADC, apparent diffusion coefficient; *r*, internal airway radius; *R*, external airway radius. NS-01: female, 77 years old; FEV1%_pred_ = 91; VDP = 1.5%; ADC = 0.24 cm^2^/sec; RA_950_ = 0.75%. NS-15: female, 69 years old; FEV1%_pred_ = 91; VDP = 2.4%; ADC = 0.20 cm^2^/sec; RA_950_ = 0.44%. EnE-03: male, 62 years old; FEV1%_pred_ = 70; VDP = 9.1%; ADC = 0.29 cm^2^/sec; RA_950_ = 4.0%. E-02: male, 79 years old; FEV1%_pred_ = 126; VDP = 16%; ADC = 0.42 cm^2^/sec; RA_950_ = 11%.

Figure2 shows the center coronal ^3^He ventilation, *ADC*, and internal (*r*) and external (*R*) acinar duct radii maps for two representative never-smokers and two ex-smokers. The same binary masks were applied for calculation of ADC and morphometry maps. The morphometry approach is complex and fitting does not always converge for all pixels and this, in some circumstances leads to voids in the morphometry maps. For the two never-smokers there was homogeneous ventilation and the ADC and morphometry maps were also regionally homogeneous with very similar mean values across the entire lung. The global mean value of the free diffusion coefficient *D*_0_ was 0.84 cm^2^/sec. For the two older ex-smokers, and especially Subject SE-02, there was visual evidence of patchy ventilation with ventilation defects obvious in the peripheral lung, as described previously (Mathew et al. 2008).

Table2 shows MRI measurements of tissue integrity (ADC) and MRI alveolar morphometry estimates as well as RA_950_, a well-understood CT measurement of emphysema for all subjects, and the never- and ex-smoker subgroups. Table S2 (online supplement) provides a subject listing of these data. As shown in Table2, ^3^He ventilation defect percent (VDP), diffusion (*ADC*, and *D*_T_) and two morphometry estimates (*r*, *h)*, as well as CT-derived RA_950_ were significantly different between never-smokers and smokers. The mean *D*_L_ and *D*_T_ estimates for never-smokers and smokers indicated that conditions *b*_max_ (*D*_L_ − *D*_T_) > 1 and *b*_max_ *D*_T_ > 1 were satisfied for the maximum *b* value (6.4 sec/cm^2^) used. In addition, as compared to literature reported values for young never-smokers, the elderly never-smokers investigated here reported greater ADC values (Fain et al. 2005; Altes et al. 2006), acinar duct radius, and mean linear intercept (Quirk et al. 2015).

**Table 2:** ^3^He MRI and CT measurements

Parameter (±SD)	Never-smokers (*n* = 38)	Ex-smokers (*n* = 21)	Significant differences (*P* value)
VDP (%)	2 (1)	14 (11)	**0.001**
ADC (cm^2^/sec)	0.23 (0.03)	0.32 (0.08)	**0.001**
*D*_L_ (cm^2^/sec)	0.53 (0.06)	0.58 (0.19)	0.2
*D*_T_ (cm^2^/sec)	0.12 (0.02)	0.44 (0.10)	**<0.0001**
*R* (*μ*m)	340 (16)	370 (48)	0.1
*r* (*μ*m)	220 (24)	260 (48)	**0.01**
*h* (*μ*m)	120 (12)	100 (7)	**<0.0001**
*L*_*m*_ (*μ*m)	260 (27)	320 (72)	0.06
*S/V* (per cm)	150 (16)	130 (28)	0.08
RA_950%_	0.68 (0.78)	7 (7)	**0.007**

Significant differences (*P* value) generated using a two-tailed *t*-test and corrected using the Holm–Bonferroni method. Bold values denotes significant difference (*P* < 0.05).

VDP, ventilation defect percent; ADC, apparent diffusion coefficient; *R*, external airway radius; *r*, internal airway radius; *h*, alveolar sheath; *L*_m_, mean linear intercept; *S/V*, surface area-to-volume ratio; RA_950_, relative area of the CT density histogram less than −950 Hounsfield units.

**Figure 3:**
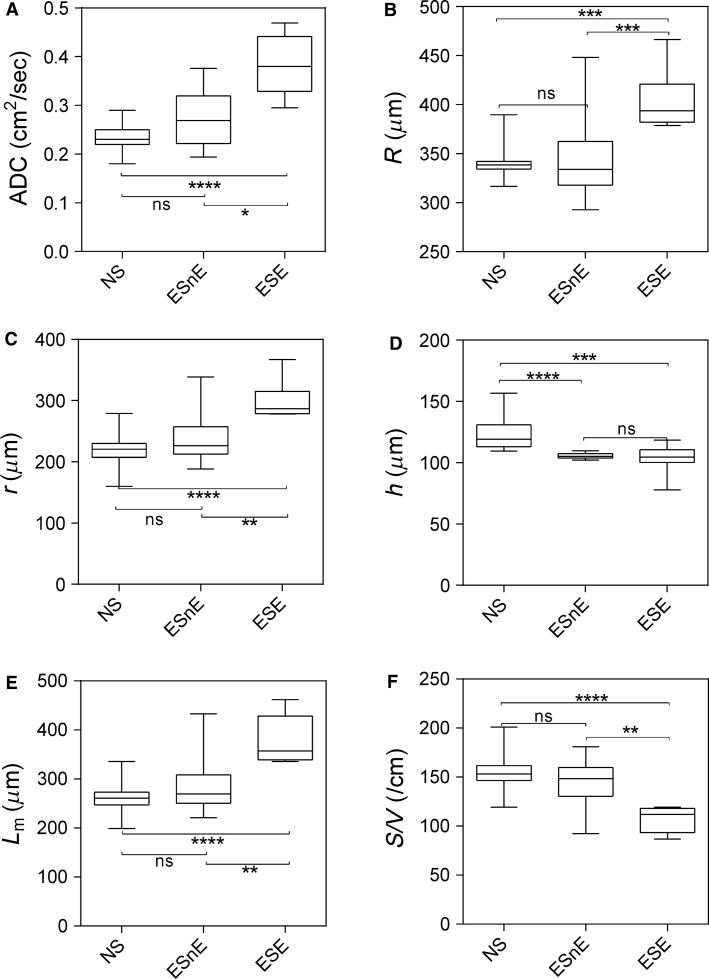
^3^He MRI morphometry measurements. Box and whisker plots show 25–75 percentile as well as minimum and maximum values. NS = never-smokers (*n* = 38); E=ex-smokers with emphysema (*n* = 8); EnE = ex-smokers with no emphysema (*n* = 13); ADC, apparent diffusion coefficient; *R*, external airway radius; *r*, internal airway radius; *h*, alveolar sheath; *L*_*m*_, mean linear intercept; *S/V*, surface area-to-volume ratio. Significant differences (*P* value) computed using a Kruskal–Wallis test with Dunn’s correction. ns = not significant *P *>* *0.05; *P < 0.05; **P < 0.01; ***P < 0.001; ****P < 0.0001.

Figure3 shows some of these comparisons in more detail. There were significant differences for never-smokers compared to ex-smokers with CT evidence of emphysema for all morphometric parameters and ADC (all *P *<* *0.001). Never-smokers were also significantly different than ex-smokers without emphysema for *h* but not for *ADC*, *R*, *r*, *h*, *L*_m_, or *S/V*. In contrast, ex-smokers with emphysema reported significantly different ADC, *R*, *r*, *L*_*m*_, *S/V* but not *h*, compared to ex- smokers without emphysema.

### Relationships with FEV_1_/FVC and DL_CO_

Pearson correlation coefficients (Bonferroni-corrected *P* values) for ^3^He morphometry measurements with FEV_1_/FVC and DL_CO_ are shown in Table3. For all subjects, there were relationships for DL_CO_ and FEV_1_/FVC with external and internal radius, mean linear intercept, ADC, RA_950_, and surface area-to-volume ratio, but only FEV_1_/FVC significantly correlated with h. For never-smokers, there were no significant correlations between the morphometry measurements and either FEV_1_/FVC or DL_CO_. In contrast, for ex-smokers, the external airway radius (*r* = −0.54, *P* = 0.04), ADC (*r* = −0.74, *P* = 0.0008), RA_950_ (*r* = −0.87, *P* < 0.0001), and surface area-to-volume ratio (*r* = 0.58, *P* = 0.03) significantly correlated with FEV_1_/FVC and there were similar significant relationships with DL_CO._

**Table 3:** Relationship of ^3^He MRI morphometry with pulmonary function measurements

Pearson correlation coefficient *R* (*P* value)						
Parameter	All (*n* = 59)		Never-smokers (*n* = 38)		Ex-smokers (*n* = 21)	
	FEV_1_/FVC	DL_CO_*	FEV_1_/FVC	DL_CO_†	FEV_1_/FVC	DL_CO_‡
*R* (*μ*m)	**−0.53 (<0.0001)**	**−0.47 (0.0006)**	**−**0.12 (0.47)	**−**0.11 (1)	**−0.54 (0.04)**	**−0.62 (0.02)**
*r* (*μ*m)	**−0.60 (<0.0001)**	**−0.48 (0.0008)**	**−**0.27 (0.51)	**−**0.08 (1)	**−**0.51 (0.05)	**−0.57 (0.03)**
*h* (*μ*m)	**0.44 (0.0005)**	0.23 (0.09)	0.38 (0.13)	**−**0.003 (1)	**−**0.21 (0.4)	**−**0.29 (0.2)
*L*_*m*_ (*μ*m)	**−0.60 (<0.0001)**	**−0.48 (0.0009)**	**−**0.24 (0.56)	**−**0.09 (1)	**−**0.49 (0.4)	**−0.53 (0.03)**
*ADC* (cm^2^/sec)	**−0.77 (<0.0001)**	**−0.67 (<0.0001)**	**−**0.24 (0.47)	**−**0.30 (0.5)	**−0.74 (0.0008)**	**−0.77 (0.0006)**
*RA*_950%_	**−0.84 (<0.0001)**	**−0.59 (<0.0001)**	**−**0.18 (0.56)	**−**0.18 (1)	**−0.87 (<0.0001)**	**−0.65 (0.01)**
*S*/*V* (per cm)	**0.62 (<0.0001)**	**0.46 (0.0007)**	0.31 (0.35)	0.06 (1)	**0.58 (0.03)**	**0.58 (0.03)**

*P* value generated using a two-tailed *t*-test and corrected using the Holm–Bonferroni method. Bold values denotes significant difference (*P* < 0.05).

FEV_1_, forced expiratory volume in 1 sec; FVC, forced vital capacity; DL_CO_, diffusing capacity of the lung for carbon monoxide percent predicted*; R*, external airway radius; *r*, internal airway radius; *h*, alveolar sheath; *L*_*m*_, mean linear intercept; *ADC*, apparent diffusion coefficient; *RA*_950_, relative area less than −950 HU; *S/V*, surface area-to-volume ratio.

* *n* = 56

† *n* = 36

‡ *n* = 20.

## Discussion

To better understand the changes in the lung parenchyma that accompany aging, we generated and evaluated noninvasive in vivo MRI estimates of acinar duct and alveolar dimensions in elderly never-smokers and smokers. We made the following observations: (1) elderly never-smokers reported diminished internal airway radius and greater alveolar depth compared to elderly ex-smokers; (2) ex-smokers with and without emphysema were significantly different for ADC, external and internal airway radius, mean linear intercept, and surface area-to-volume ratio but not alveolar depth; (3) there was a significant difference for alveolar depth for never-smokers and ex-smokers without emphysema, in whom all other morphological measures and ADC were not significantly different; and (4) in elderly never-smokers, there were no significant correlations, whereas in elderly ex-smokers, FEV_1_/FVC and DL_CO_ significantly correlated with ADC, RA_950_, *R*, and *S/V*, while DL_CO_ also significantly correlated with *r* and *L*_m_.

### Differences between never- and ex-smokers

As expected, the vast majority of elderly never-smokers reported normal pulmonary function measurements. While two never-smokers reported FEV_1_/FVC values less than (but very close to) the GOLD threshold for COPD, there were no occupational or second hand smoke exposures that could explain these findings. When compared to literature reported values for younger never-smokers, we observed elevated ADC (0.23 cm^2^/s vs. 0.17 cm^2^/s) (Fain et al. 2006), diminished alveolar depth (120 vs. 130* μ*m), and elevated *L*_m_ (260 vs. 240* μ*m) (Quirk et al. 2015) in the elderly never-smokers. These findings may be attributed to a loss of elastin or change in collagen content, organization, and/or distribution with age (West 1971; Sobin et al. 1988). Moreover, these differences may also be explained by inflammation/edema or fibrosis, although there is no evidence to suggest this in any of the subgroups studied here.

**Figure 4:**
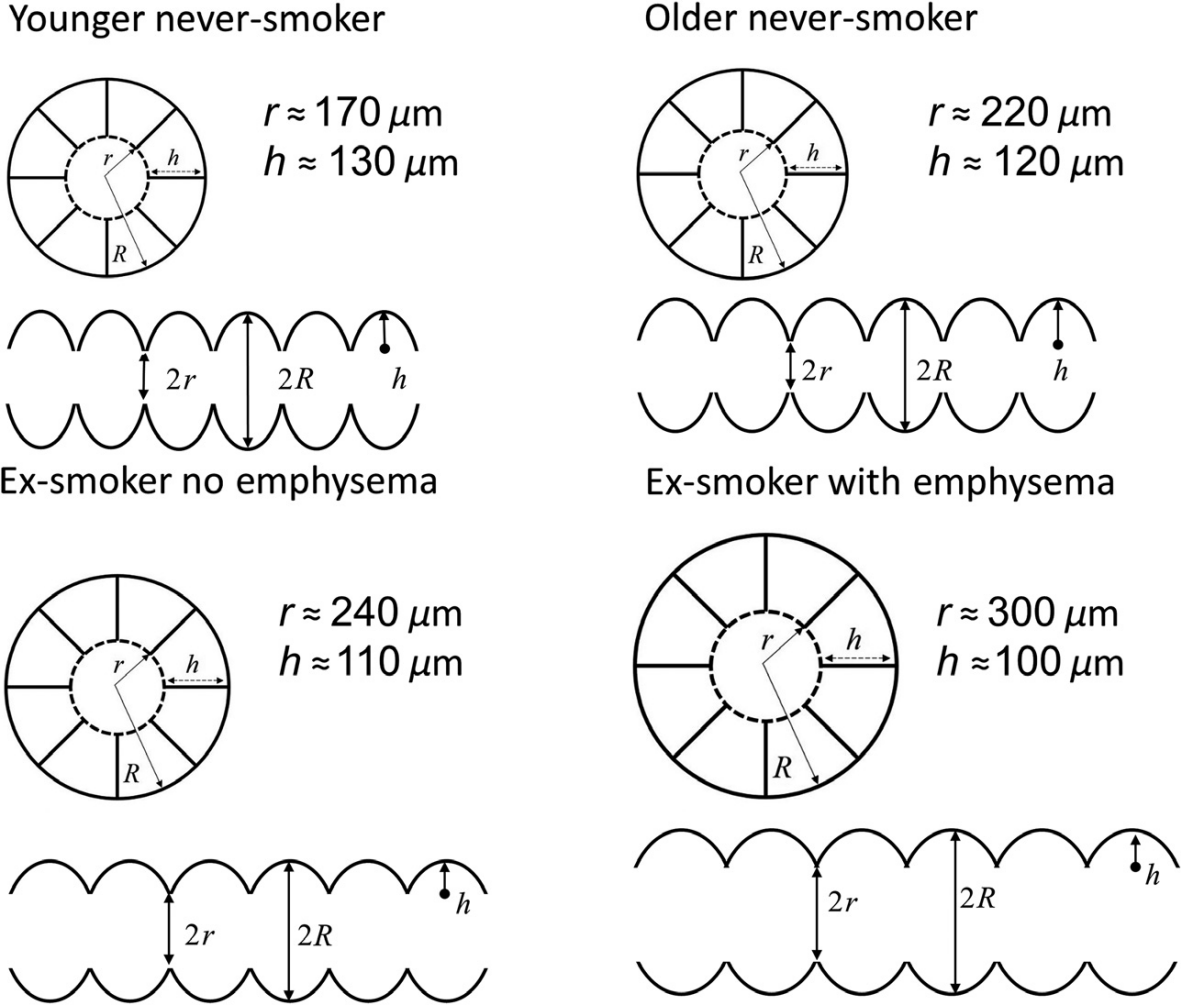
Alveolar duct schematic. Acinar duct and alveolar morphological parameters were based on Weibel model (Weibel 1963) for acinar duct parameters shown for a representative young never-smoker (Quirk et al. 2015), elderly never-smoker, and elderly never-smoker with and without emphysema.

The acinar morphometric measurements in elderly never-smokers were also significantly different compared to the ex-smokers with emphysema, but when compared to ex-smokers without emphysema, only alveolar depth was significantly different. The finding of elevated ADC and some morphometric measures in ex-smokers was also supported by the finding of abnormally elevated RA_950_ measurements. As shown in schematic in Figure4, elderly ex-smokers reported diminished alveolar depth and *S/V* as well as greater *L*_m_ as compared to never-smokers. There are several potential mechanisms for these findings including the fact that emphysematous enlargement of the acinar ducts can result in flattening of the alveolus and retraction of the alveolar septa (Hartroft 1945).

It is also important to point out that there were differences between ex-smokers with and without emphysema and the elderly never-smokers. While ADC, external radius, internal radius, mean linear intercept, and surface area-to-volume ratio differed were different between ex-smokers with and without emphysema, this was not the case for never-smokers and the ex-smokers without emphysema in whom only alveolar depth differed. We observed no significant difference in alveolar depth between ex-smokers with (mean h = 100 *μ*m) and without (mean h = 110 *μ*m) emphysema; this diminished h value was not sufficient to detect significant differences between the two groups of ex-smokers. There were, however, significant differences in ADC and all other morphometric measurements.

We must acknowledge the sample sizes for ex-smokers with and without emphysema are different and this is a study limitation. However, we thought it was important to point out the heterogeneity of emphysema in these volunteers. Although this is a very small subgroup to evaluate, it helps explain parenchyma differences due to lung aging relative to smoking; these comparisons also provide motivation for a larger study in ex-smokers with COPD. We think these are important clues that point to the differences between senile emphysema and the mild emphysema that is coincident with tobacco smoke exposure.

### Relationships between morphometric and pulmonary function measurements

In all participants, there were moderate and strong relationships between all morphological measurements, RA_950_ and ADC with FEV_1_/FVC, wheras DL_CO_ had no relationship with h but related to all other morphological measurements, RA_950_, and ADC, and this provides a way to internally validate the MRI estimates and their clinical relevance. In the elderly never-smokers, neither FEV_1_/FVC nor DL_CO_ had any significant relationship with ADC, RA_950_, or any of the morphological measurements. The lack of significant relationships between acinar morphological estimates and DL_CO_ in elderly never-smokers was not surprising, but the finding of significantly different morphometric measurements in the presence of normal DL_CO_ was somewhat unexpected and may underscore the sensitivity of MRI to acinar duct geometries. At the same time, normal RA_950_ values in the presence of “elevated” *L*_m_ may also provide strong evidence of the sensitivity of MRI to very mild emphysema even when CT is normal. The mean *L*_m_ value obtained for older never-smokers was 5% larger than reported previously (240–250 *μ*m) (Quirk et al. 2015), this may be due to the 10-year age difference between these subjects. The elevated mean *L*_m_ value may reflect normal lung aging (a loss of elastin or change in collagen content) over the 10-year period.

### Considerations and limitations

We must acknowledge a number of study limitations including the fact that ^3^He MRI requires unique expertise and equipment. There is an extremely limited supply of helium gas, making further follow-up studies and clinical implementation of this technique unlikely. One common limitation of the multiple *b*-value method is the relatively long data acquisition time (i.e., longer than typical breath-hold durations) and typically lower SNR compared to the more commonly used two *b*-value ADC method (due to the larger number of RF pulses). To overcome these inherent limitations, parallel imaging was previously piloted in asthma patients (Chang et al. 2015). This previous work was similar to our approach, whereby 3D whole-lung morphometry data were acquired based on 5 *b* values during a single 15-sec breath-hold, with adequate spatial resolution (Chang et al. 2015). By combining our approach with parallel imaging, further reductions in acquisition time and improved spatial resolution and/or SNR can be achieved.

It is also important to acknowledge that there is still room for improvement in the computational modeling of pulmonary morphometry. Algorithm optimization is still required but MRI is more time efficient and less invasive than lung stereology. However, MRI morphometry still requires manual observer interaction and is computationally intense. Moreover, although the morphological equations we used were appropriate for healthy and mildly emphysematous lungs, when acinar morphologies deviate significantly from these structures, as in the case of severe emphysema or bronchopulmonary dysplasia, the “cylindrical” (Yablonskiy et al. 2002) and “branching” models (Parra-Robles et al. 2010; Parra-Robles and Wild 2012) may not be appropriate. The morphometry model also has limitations, but to our knowledge this is the only available model in the literature providing mathematical equations for the extraction of lung microstructure parameters. Using this model, the correspondence between MRI-based *L*_m_ estimates and the histological mean linear intercept (MLI) estimates was also confirmed previously (Yablonskiy et al. 2014). Other limitations originating from the morphometry model itself were also described previously (Parra-Robles et al. 2010; Parra-Robles and Wild 2012). We compared diffusion MRI measurements obtained at 3 T and 1.5 T because previously published work suggested that ADC values and morphometric parameters may be overestimated at higher magnetic fields (Parra-Robles et al. 2012a). To investigate the effect of field strength we conducted a substudy in young never-smokers (mean age = 22 years, data not shown) and obtained ADC values for five of these never-smokers and morphometric parameters for one of them. The ADC estimates at 3 T (mean = 0.172 cm^2^/s) were in agreement with ADC values (mean = 0.178 cm^2^/s) (Fain et al. 2006) obtained at 1.5 T using ∆ = 1.46 msec and the same diffusion gradient waveform and scanner platform (GEHC, trapezoidal pulses with 500 *μ*sec ramp times, 460 *μ*sec peak pulse width, and 1.94848 G/cm peak pulse amplitude, *b* = 1.6 sec/cm^2^). In addition, the morphometry results for a single young subject (*L*_m_ = 195 *μ*m) was within the range of *L*_m_ values (180–220 *μ*m) observed at 1.5 T (Quirk et al. 2015).

While generally smaller ADC values for never-smokers were reported at 1.5 T (Swift et al. 2005) using the same diffusion time (1.46 msec) and diffusion gradient waveform, there previous results are not directly comparable to what we report because these data stem from younger never-smokers (mean = 52 years of age) and additionally, the b value was almost twice as large (2.86 sec/cm^2^). Both of these issues would influence ADC to lower values. At the same time, the ADC for never-smokers were in agreement with previously published values obtained at 1.5 T (Fain et al. 2006). Another limitation stems from the assumption at the foundation of the diffusion/morphometry relationship which presumes that the diffusing gas atoms cannot penetrate through alveolar walls. Alveolar walls have pores, although their effects on *D*_*L*_ and *D*_*T*_ are considered negligible in healthy adults due to the small number of microscopic (<10 *μ*m) pores present (Nagai et al. 1995). In elderly and emphysematous lung, however, this assumption likely weakens, as more pores of variable size (>20 *μ*m) are present, ultimately increasing the transverse and longitudinal ADC values (Nagai and Thurlbeck 1991). Another drawback stems from the fact that an enormous amount of data is reduced to a few parameters of an extremely simplified whole-lung average anatomic model of the acini and that valuable information on gross differences in topographical heterogeneity may be neglected. In this regard, we note however that gross differences in topographical heterogeneity were explored using CT and we did not find such gross in these participants – though this is a common feature in more advanced COPD. Finally, it should be noted that a diffusion time of 1.46 msec was used in order to enable comparisons with previous measurements made in the same subjects. Based on theoretical predictions (Yablonskiy et al. 2009), the optimal diffusion time in human lungs using ^3^He MRI is 1.6–1.8 msec and therefore the use of a smaller diffusion time may lead to overestimates of morphometric parameters (Parra-Robles et al. 2012b). Nevertheless, any potential overestimation of *R* and *r* due to the 8% smaller *∆* would be quite minor and not change the conclusions of this study.

## Conclusions

This is the first study to implement noninvasive in vivo MRI morphometry in a relatively large group of elderly volunteers with and without a history of tobacco smoking that aimed to provide a better understanding of the parenchyma changes that accompany lung aging and smoking. This study showed that there are significant but small differences in never- and ex-smokers in acinar duct internal radius and alveolar depth and demonstrated the sensitivity of MRI noninvasive measurements of pulmonary microstructure to dissect the effects of smoking and aging on acinar morphometry.

## Supporting Information

**Table S1.** Subject listing of demographics. **Table S2.** Subject listing of MRI morphometry data.
