# Supplementary material for: Noninvasive quantification of alveolar morphometry in elderly never- and ex-smokers
Source: Physiol Rep. 2015 Oct 13;3(10):e12583. doi: 10.14814/phy2.12583 (PMC4632953; doi:10.14814/phy2.12583)
Supplement: Supplementary file 1 [file phy20003-e12583-sd1.docx]

**Table 1S. (Online Supplement) Subject Listing of Demographics**

| Subject | Sex | Age  (Yr) | BMI  (kg/m^2^) | FEV_1_  (%pred) | FVC  (%pred) | FEV_1_/FVC  (%) | RV  (%pred) | TLC  (%pred) | RV/TLC  (%pred) | DLco  (%pred) | RA_950_  (%) |
| --- | --- | --- | --- | --- | --- | --- | --- | --- | --- | --- | --- |
| E-01 | M | 80 | 29 | 105 | 118 | 63 | 130 | 117 | 110 | 84 | 8.3 |
| E-02 | M | 79 | 27 | 126 | 142 | 63 | 115 | 126 | 91 | 62 | 10.5 |
| E-03 | M | 84 | 24 | 99 | 107 | 63 | 94 | 90 | 106 | 59 | 8.5 |
| E-04 | M | 75 | 26 | 30 | 61 | 36 | 260 | 134 | 192 | 31 | 26.4 |
| E-05 | F | 72 | 28 | 56 | 83 | 51 | 132 | 111 | 118 | 59 | 7.9 |
| E-06 | M | 84 | 23 | 52 | 81 | 44 | 169 | 113 | 153 | 47 | 23.8 |
| E-07 | M | 70 | 29 | 47 | 84 | 41 | 142 | 100 | 137 | 62 | 13.8 |
| E-08 | M | 71 | 21 | 60 | 78 | 57 | 133 | 95 | 137 | 39 | 8.0 |
| E Mean (±SD) | **7M/1F** | **77 (6)** | **26 (3)** | **72 (34)** | **94 (26)** | **52 (11)** | **150 (50)** | **110 (15)** | **130 (32)** | **55 (16)** | **13 (8)** |
| EnE-01 | F | 53 | 36 | 101 | 101 | 78 | 92 | 104 | 89 | 83 | 1.5 |
| EnE-02 | F | 79 | 24 | 123 | 133 | 68 | 128 | n/a | n/a | n/a | 5.4 |
| EnE-03 | M | 62 | 28 | 70 | 88 | 60 | 134 | 104 | 127 | 59 | 4.0 |
| EnE-04 | F | 71 | 31 | 92 | 98 | 71 | 86 | 108 | 80 | 60 | 2.8 |
| EnE-05 | F | 71 | 41 | 85 | 92 | 71 | 97 | 96 | 101 | 73 | 3.7 |
| EnE-06 | F | 67 | 36 | 110 | 106 | 79 | 89 | 100 | 90 | 74 | 1.0 |
| EnE-07 | M | 68 | 28 | 126 | 121 | 77 | 134 | 120 | 112 | 96 | 2.9 |
| EnE-08 | M | 55 | 30 | 67 | 67 | 76 | 94 | 77 | 122 | 115 | 2.5 |
| EnE-09 | F | 62 | 26 | 78 | 97 | 62 | 105 | 101 | 105 | 98 | 4.7 |
| EnE-10 | M | 56 | 27 | 88 | 81 | 82 | 90 | 85 | 104 | 76 | .48 |
| EnE-11 | M | 57 | 20 | 91 | 119 | 58 | 119 | 120 | 97 | 87 | 4.0 |
| EnE-12 | M | 69 | 24 | 89 | 94 | 70 | 107 | 98 | 110 | 67 | .83 |
| EnE-13 | M | 85 | 28 | 140 | 130 | 74 | 122 | 111 | 112 | 94 | 1.8 |
| EnE Mean (±SD) | **7M/6F** | **66 (10)** | **30 (6)** | **97 (22)** | **102 (19)** | **71 (8)** | **106 (17)** | **102 (13)** | **104 (14)** | **82 (17)** | **3 (2)** |
| E Mean (±SD) | **14M/7F** | **70 (10)** | **28 (5)** | **87 (29)** | **99 (22)** | **64 (13)** | **122 (39)** | **105 (14)** | **114 (26)** | **71 (21)** | **7 (7)** |
| NS-01 | F | 77 | 26.3 | 91 | 94 | 72 | 144 | 117 | 125 | 107 | .74 |
| NS-02 | F | 71 | 25.3 | 103 | 101 | 78 | 84 | 97 | 85 | 86 | .14 |
| NS-03 | M | 72 | 27.6 | 90 | 89 | 74 | 98 | 90 | 106 | 92 | .20 |
| NS-04 | M | 79 | 24.6 | 142 | 136 | 74 | 47 | 110 | 44 | 103 | .62 |
| NS-05 | M | 68 | 22.6 | 88 | 95 | 69 | 103 | 94 | 106 | 107 | .47 |
| NS-06 | F | 67 | 29.7 | 91 | 74 | 94 | 76 | 80 | 96 | 78 | .09 |
| NS-07 | F | 67 | 21.1 | 98 | 98 | 76 | 120 | 111 | 108 | 85 | 1.7 |
| NS-08 | F | 69 | 29.4 | 115 | 110 | 77 | 117 | 116 | 101 | 113 | .54 |
| NS-09 | F | 63 | 20.9 | 109 | 105 | 80 | 110 | 110 | 98 | 103 | .19 |
| NS-10 | F | 83 | 19.5 | 132 | 136 | 71 | 106 | 118 | 90 | 89 | 1.0 |
| NS-11 | M | 78 | 30.7 | 110 | 101 | 78 | 89 | 95 | 91 | 84 | 1.0 |
| NS-12 | F | 67 | 21.4 | 124 | 111 | 85 | 70 | 97 | 73 | 87 | .17 |
| NS-13 | M | 68 | 24.4 | 95 | 94 | 75 | 123 | 102 | 116 | 108 | .18 |
| NS-14 | M | 86 | 24.2 | 100 | 76 | 88 | 58 | 62 | 94 | 74 | .25 |
| NS-15 | F | 69 | 30.2 | 91 | 89 | 77 | 82 | 91 | 92 | 148 | .44 |
| NS-16 | M | 73 | 29.7 | 106 | 102 | 75 | 91 | 95 | 94 | 73 | 1.2 |
| NS-17 | F | 71 | 27.4 | 109 | 108 | 76 | 103 | 109 | 93 | 76 | .06 |
| NS-18 | M | 82 | 27.1 | 77 | 86 | 64 | 104 | 90 | 112 | 89 | .51 |
| NS-19 | M | 71 | 26.6 | 125 | 117 | 78 | 110 | 117 | 92 | 81 | .92 |
| NS-20 | F | 76 | 30.5 | 106 | 102 | 78 | 102 | 99 | 102 | 74 | .59 |
| NS-21 | M | 79 | 24.1 | 111 | 109 | 73 | 66 | 91 | 73 | 80 | 1.6 |
| NS-22 | F | 80 | 28.6 | 115 | 119 | 71 | 95 | 102 | 97 | 86 | 2.6 |
| NS-23 | F | 73 | 32.1 | 112 | 104 | 80 | 85 | 96 | 88 | 86 | .32 |
| NS-24 | F | 63 | 25.5 | 94 | 89 | 81 | 90 | 95 | 92 | 113 | .48 |
| NS-25 | F | 67 | 25.9 | 88 | 84 | 79 | 45 | 76 | 59 | 81 | .06 |
| NS-26 | F | 79 | 31.7 | 117 | 111 | 78 | 95 | 102 | 92 | n/a | .55 |
| NS-27 | F | 74 | 26.2 | 111 | 106 | 78 | 88 | 99 | 90 | n/a | .32 |
| NS-28 | F | 64 | 24 | 108 | 103 | 80 | 87 | 104 | 84 | 99 | .14 |
| NS-29 | F | 68 | 21.8 | 117 | 114 | 78 | 110 | 116 | 94 | 75 | .32 |
| NS-30 | F | 77 | 34.3 | 104 | 105 | 74 | 105 | 104 | 103 | 68 | .57 |
| NS-31 | F | 63 | 25.5 | 106 | 101 | 80 | 88 | 99 | 87 | 80 | .17 |
| NS-32 | F | 80 | 26.6 | 108 | 110 | 73 | 100 | 103 | 96 | 101 | .34 |
| NS-33 | M | 81 | 25.4 | 119 | 98 | 85 | 91 | 84 | 112 | 63 | .19 |
| NS-34 | F | 78 | 23.7 | 129 | 114 | 85 | 123 | 120 | 103 | 82 | 2.4 |
| NS-35 | M | 84 | 23.9 | 155 | 132 | 80 | 96 | 101 | 98 | 73 | 1.4 |
| NS-36 | M | 69 | 31.1 | 79 | 80 | 73 | 114 | 92 | 123 | 101 | .26 |
| NS-37 | M | 71 | 24.2 | 126 | 119 | 78 | 120 | 122 | 96 | 98 | .32 |
| NS-38 | M | 75 | 26.3 | 123 | 116 | 76 | 92 | 102 | 92 | 81 | 3.5 |
| NS Mean (±SD) | **15M/23F** | **73 (6)** | **26 (3)** | **108 (17)** | **104 (15)** | **77 (6)** | **95 (21)** | **100 (13)** | **95 (16)** | **90 (17)** | **.7 (.8)** |

**Table 2S. (Online Supplement) Subject listing of MRI morphometry Data**

| Subject | ADC  (cm^2^/s) | R  (µm) | r  (µm) | h  (µm) | L_m_  (µm) | S/V  (cm^-1^) |
| --- | --- | --- | --- | --- | --- | --- |
| E-01 | 0.32 | 380 | 300 | 78 | 430 | 93 |
| E-02 | 0.42 | 470 | 370 | 100 | 460 | 90 |
| E-03 | 0.36 | 390 | 280 | 110 | 340 | 120 |
| E-04 | 0.47 | 400 | 280 | 110 | 340 | 120 |
| E-05 | 0.30 | 380 | 280 | 100 | 340 | 120 |
| E-06 | 0.45 | 390 | 290 | 100 | 360 | 110 |
| E-07 | 0.36 | 400 | 280 | 120 | 360 | 110 |
| E-08 | 0.40 | 430 | 320 | 110 | 420 | 95 |
| E Mean (±SD) | **0.38 (0.08)** | **400 (30)** | **300 (31)** | **100 (12)** | **380 (49)** | **110 (13)** |
| EnE-01 | 0.19 | 290 | 190 | 100 | 220 | 180 |
| EnE-02 | 0.39 | 450 | 340 | 110 | 430 | 90 |
| EnE-03 | 0.29 | 340 | 240 | 100 | 290 | 140 |
| EnE-04 | 0.38 | 320 | 210 | 110 | 270 | 150 |
| EnE-05 | 0.28 | 340 | 230 | 110 | 270 | 150 |
| EnE-06 | 0.27 | 340 | 240 | 100 | 280 | 140 |
| EnE-07 | 0.21 | 290 | 190 | 100 | 220 | 180 |
| EnE-08 | 0.22 | 320 | 220 | 100 | 260 | 160 |
| EnE-09 | 0.23 | 320 | 210 | 110 | 250 | 160 |
| EnE-10 | 0.22 | 320 | 210 | 110 | 250 | 160 |
| EnE-11 | 0.35 | 380 | 270 | 110 | 330 | 120 |
| EnE-12 | 0.26 | 390 | 290 | 110 | 350 | 120 |
| EnE-13 | 0.28 | 330 | 230 | 110 | 270 | 150 |
| EnE Mean (±SD) | **0.27 (0.06)** | **340 (43)** | **240 (52)** | **110 (2)** | **280 (57)** | **150 (25)** |
| NS-01 | .24 | 340 | 220 | 120 | 260 | 150 |
| NS-02 | .24 | 380 | 270 | 120 | 320 | 130 |
| NS-03 | .23 | 370 | 2300 | 130 | 280 | 140 |
| NS-04 | .25 | 380 | 260 | 120 | 320 | 130 |
| NS-05 | .21 | 330 | 210 | 120 | 250 | 160 |
| NS-06 | .18 | 320 | 160 | 160 | 200 | 200 |
| NS-07 | .23 | 330 | 210 | 120 | 250 | 160 |
| NS-08 | .22 | 340 | 230 | 110 | 270 | 150 |
| NS-09 | .19 | 330 | 170 | 150 | 210 | 190 |
| NS-10 | .22 | 340 | 220 | 120 | 260 | 150 |
| NS-11 | .26 | 340 | 230 | 110 | 270 | 150 |
| NS-12 | .19 | 330 | 200 | 140 | 240 | 170 |
| NS-13 | .23 | 340 | 210 | 120 | 250 | 160 |
| NS-14 | .23 | 340 | 230 | 110 | 270 | 150 |
| NS-15 | .20 | 340 | 210 | 130 | 250 | 160 |
| NS-16 | .29 | 390 | 280 | 110 | 340 | 120 |
| NS-17 | .20 | 340 | 190 | 140 | 230 | 170 |
| NS-18 | .25 | 340 | 230 | 110 | 270 | 150 |
| NS-19 | .23 | 330 | 210 | 120 | 250 | 160 |
| NS-20 | .20 | 330 | 190 | 140 | 230 | 170 |
| NS-21 | .27 | 340 | 230 | 110 | 270 | 150 |
| NS-22 | .24 | 340 | 230 | 110 | 280 | 150 |
| NS-23 | .25 | 340 | 210 | 130 | 250 | 160 |
| NS-24 | .22 | 340 | 230 | 120 | 270 | 150 |
| NS-25 | .22 | 340 | 200 | 140 | 230 | 170 |
| NS-26 | .22 | 340 | 230 | 110 | 280 | 150 |
| NS-27 | .23 | 340 | 230 | 110 | 270 | 150 |
| NS-28 | .25 | 340 | 230 | 120 | 270 | 150 |
| NS-29 | .24 | 340 | 210 | 120 | 250 | 160 |
| NS-30 | .23 | 340 | 230 | 110 | 270 | 150 |
| NS-31 | .22 | 330 | 200 | 130 | 240 | 170 |
| NS-32 | .23 | 330 | 220 | 120 | 260 | 160 |
| NS-33 | .25 | 340 | 220 | 120 | 260 | 160 |
| NS-34 | .27 | 390 | 270 | 120 | 320 | 130 |
| NS-35 | .26 | 340 | 230 | 110 | 270 | 150 |
| NS-36 | .22 | 330 | 200 | 130 | 230 | 170 |
| NS-37 | .23 | 330 | 210 | 130 | 250 | 160 |
| NS-38 | .29 | 380 | 270 | 110 | 320 | 120 |
| NS Mean (±SD) | **0.23 (0.03)** | **340 (18)** | **220 (25)** | **120 (12)** | **260 (30)** | **150 (17)** |
